# A High-Performance Polymer Composite Column for Coronavirus Nucleic Acid Purification

**DOI:** 10.21203/rs.3.rs-3261727/v1

**Published:** 2023-08-25

**Authors:** Akli Zarouri, Aaron M. T. Barnes, Hamada Aboubakr, Vinni Thekkudan Novi, Qiuchen Dong, Andrew Nelson, Sagar Goyal, Abdennour Abbas

**Affiliations:** University of Minnesota, Twin Cities; University of Minnesota, Twin Cities; University of Minnesota, Twin Cities; University of Minnesota, Twin Cities; University of Minnesota, Twin Cities; University of Minnesota, Twin Cities; University of Minnesota, Twin Cities; University of Minnesota, Twin Cities

**Keywords:** Nucleic acid purification, Polymer composite, Coronavirus, RT-PCR detection, spin columns

## Abstract

Here, we report the development of a novel polymer composite (PC) purification column and kit. The performance of the PC columns was compared to conventional silica gel (SG) columns for the purification of nucleic acids from coronaviruses, including SARS-CoV-2, in 82 clinical samples. The results shows that PC-based purification outperforms silica gel (SG)-based purification by enabling a higher sensitivity (94%), accuracy (97%), and by eliminating false positives (100% selectivity). The high selectivity is critical for efficient patient triage and resource management during pandemics. Furthermore, PC-based purification exhibits three times higher analytical precision than a commonly used SG-based nucleic acid purification thereby enabling a more accurate quantification of viral loads and higher reproducibility.

## Introduction

One of the critical tools in the fight against the ongoing COVID pandemic is the rapid and the accurate detection of the causative agent, SARS-CoV-2, which enables early prevention of outbreaks in communities and hospitals. Currently, Reverse Transcription-Polymerase Chain Reaction (RT-PCR) is the gold standard in diagnostics and pathogen detection including SARS-CoV-2.^1,2^ This technique has been recommended by The World Health Organization (WHO) as the gold standard in testing^3^. This is due to the high sensitivity and specificity compared to other viral detection methods such as viral antigen detection, standard plaque assay, serology, or CRISPR-based techniques.^4–7^ However, RT-PCR performance is inherently tied to the quality and quantity of the nucleic acid extract present in the sample Even slight variations in quality can lead to misleading results, including both false negatives and false positives^8^. The accuracy of the test is intricately linked to the efficiency of the extraction and purification processes^9^ The nucleic acid purification techniques that are currently used for this purpose suffer from low specificity (more false positives) and/or sensitivity (more false negatives). The implications of these elevated false negative rates may create considerable obstacles in effectively curbing the spread of viral infections^10^. Silica gel spin columns and magnetic beads have been commonly employed for nucleic acid extraction. Silica gel is negatively charged and the adsorption of the negatively charged nucleic acid macromolecule to the silica gel surface requires a positively charged binding agent which forms a complex with both nucleic acid and silica gel.^11^ This is facilitated by a high concentration of chaotropic salt, to which the biological samples are exposed during the nucleic acid extraction process.^12,13^ The salt acts as a bridge between the nucleic acid backbone and the silica surface by forming a layer of positive ions.^14^

Nevertheless, silica-based purification systems face limitations due to the strong binding affinity of smaller nucleic acid fragments. This compromises the overall binding efficiency to the silica matrix and renders it non-reusable. Furthermore, the extraction of these smaller fragments becomes increasingly challenging due to this strong binding interaction.^15,16^ Silica gel columns also suffer from low nucleic acid recovery rate for samples containing lower than 1 μg of total nucleic acid. In such cases, the silica gel membrane needs a carrier nucleic acid to improve the yield,^17^ which increases costs. Extraction using magnetic beads on the other hand faces challenges with interferences in PCR amplification and can be labor intensive.^15^ While labor can be reduced using automated systems, it is still difficult to use magnetic separation for samples with large volumes (> 10 mL) due to limitations in the space distribution of the electromagnetic field.^18^ Such limitations have made the testing process time consuming and costly, especially in the case of wastewater-based epidemiology.

The use of filter paper has been reported as a viable alternative to silica-based materials for nucleic acid purification from diverse sources^19^. However, it should be noted that within the paper, the accessibility of hydroxyl groups (OH) on the surface of cellulosic chains is limited, as some are inward-facing and not easily accessible^20^. Furthermore, the availability of surface OH groups on cellulose fibers typically ranges from 1–3% of the total hydroxy groups present in the original cellulose sample^21^. Consequently, the functionalization of fibers with TEOS provides a promising approach to enhance the availability of OH groups. This, in turn, enables a more efficient uptake of nucleic acids through the positive salt bridge formed between the OH groups and nucleic acid molecules.

Building on this premise, a new polymer composite filter is proposed here. The filter is based on a microporous cellulose paper functionalized with Tetraethyl orthosilicate (TEOS) and optimized for the binding and easy elution of nucleic acids. A performance comparison study between commercially available silica gel (SG) columns and extraction kits and the polymer composite (PC) columns and lab-made reagent kit is performed. The comparison was first conducted for the detection of the transmissible gastroenteritis virus (TGEV) as a lower biosafety level animal coronavirus surrogate to the human sever acute respiratory syndrome coronavirus-2 (SARS-CoV-2), followed by a full-scale study by the medical school at the University of Minnesota for the detection of SARS-CoV-2, the causative agent of COVID-19, in clinical samples.

## Results and Discussion

### RT-qPCR Detection and Quantification of TGEV

A rapid feasibility study was conducted to confirm the functionality of both the SG and PC purification methods and kits for the detection of TGEV, before pursuing a full-scale study to evaluate the analytical parameters (sensitivity, specificity, limit of detection). The results from the feasibility study show that both the SG and PC-based kits offer reliable detection and quantification of TGEV as shown in Supplementary **Figure a**. Following these positive results, more extensive studies were conducted to evaluate and compare the performance of the two nucleic acid extraction and purification methods.

### RT-qPCR Detection and Quantification of SARS-CoV-2

Preliminary and full-scale studies for the extraction and detection of SARS-CoV-2 RNA from COVID-19 patients’ samples were conducted by the COVID-19 Diagnostic Laboratory at the University of Minnesota. RT-PCR quantification of SARS-CoV-2 samples collected from infected patients was done using RNA extracted by the PC-based kit and the silica gel (SG)-based kit. The efficiency of the kits in extracting viral RNA was studied based on five analytical parameters: sensitivity, specificity, limit of detection (LOD), accuracy, and reproducibility. The preliminary study was conducted on 16 clinical samples. To comply with the FDA assay validation requirements, 32 positive samples collected from COVID-19 patients and 32 negative samples (controls) were used for the Full-scale study (Table 1).

#### Clinical Sensitivity and Specificity.

**Figure 1:**
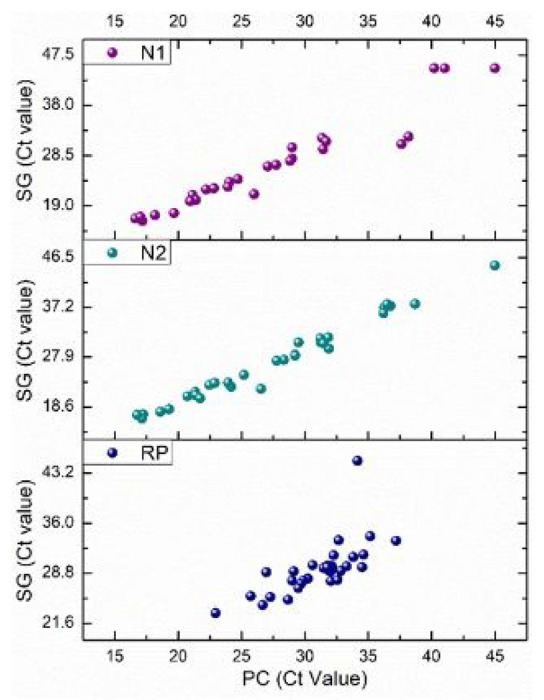
Comparison of Ct values obatined using SG- vs. PC-based purification kits for RT-qPCR detction od SARS-CoV-2.

Figure 1 shows a good linearity indicating an agreement between the Ct values obtained with SG-based kits and those obtained with PC-based kits. The sensitivity [True Positives “TP7”/ (TP + False Negatives “FN”)], specificity [True Negatives “TN”/ (TN + False Positives “FP”)], and accuracy [(TN + TP)/(TN + TP + FN + FP)] data for both kits are shown in Table 1. The results reveal that the sensitivity of detection improved by using the PC-based kit as compared to the widely used SG-based kit. Likewise, the specificity of RNA detection drastically improved up to a 100% when the PC-based kit was used as compared to the SG-based kit, which only showed around 84% specificity. This means that PC-based purification can eliminate false positives, which is crucial for patient triage during pandemics. In addition to the improved accuracy by 8%, the PC-based purification showed only a 0.89% variation in sensitivity and 0% variation in specificity between the preliminary study and the full-scale study, reflecting a high consistency in performance. However, the variations are 8% and 15.6%, respectively, for the SG-based purification between the two studies. This suggests that the PC-based kit is less susceptible to variations in samples, users, and experimental conditions. This is partly due to the higher analytical precision (ability to differentiate smaller changes in viral loads) as discussed below. The raw data on the sensitivity and specificity calculations are available in the supplementary information (**Tables a** and **b**).

#### Limit of Detection (LOD).

**Figure 2:**
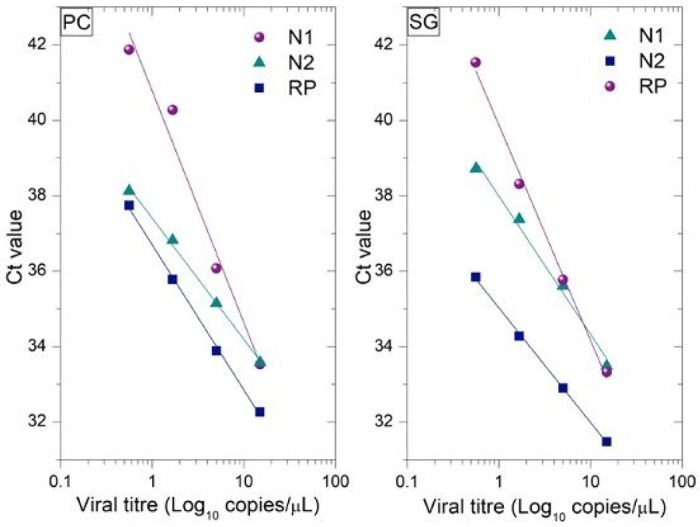
Comparison of the detection limits and Ct values of the RNA extraction kits, PC and SG in detecting SARS-CoV-2. The x axis is in the logarithmic scale. This figure is also used to calculate the analytical precision from the slope of the linear fit.

The LOD was calculated based on the RT-PCR results obtained from both the PC-based kit and the SG-based kit. The results are summarized in Fig. 2, which show that both kits exhibit similar LOD around 0.57 copy/μL and within the 5% standard deviation that is usually observed for most commercial extraction kits (Table 2). The raw data used for LOD analyses is provided in Supplementary Information (**Table c**).

Although the LOD values were similar for both kits, Fig. 2 shows a significant difference in analytical precision, corresponding to the slope of the linear fits, and reflecting the ability to distinguish smaller changes in viral loads. The results show that changes in RNA concentration by 1 copy/μL leads to a change in the Ct values by 0.28 for the PC-based purification and only 0.09 for SG-based purification (Table 2). This indicates that the PC-based kit exhibits at least three times higher analytical precision than the commonly used SG-based kit.

## Materials and Methods

### Materials and reagents

Whatman filter Paper grade 5 was purchased from Cytiva. Tetraethyl orthosilicate (TEOS, Iminodiacetic acid (IDA) 98%), Guanidine thiocyanate (GuTC) and Guanidine hydrochloride (GuHCl) were obtained from Sigma-Aldrich. Empty spin columns with O-rings and collection tubes were purchased from Shanghai Perfect Foreign trade. dsDNA assay kit was purchased from Invitrogen (Carlsbad, CA, USA). The RNA assay kit was purchased from Invitrogen (Carlsbad, CA, USA). Reagents for TGEV propagation including Eagle’s Minimum Essential Medium, Corning^®^ MEM and Dulbecco’s Modified Eagle Medium Corning^®^ DMEM were purchased from Mediatech Inc. (Manassas, VA, USA), 8% fetal bovine serum (FBS) and 1× antibiotic-antimycotic were purchased from Gibco by Life Technologies (Carlsbad, CA, USA), bovine serum albumin solution-fraction V (7.5% BSA) was purchased from Thermo Fisher Scientific (MA, USA) and 0.65 U/mL TPCK-trypsin was purchased from Worthington biochemical Inc. (NJ, USA). PCR primers and probe were manufactured by Integrated DNA Technologies (IDT Inc., IA, USA). AgPath-ID One-Step RT-PCR kit and ABI MicroAmp^®^ Fast Optical 96-Well Reaction Plates were purchased from Applied Biosystems by ThermoFisher Scientific (CA, USA). Matrix gene transcript RNA was obtained from the University of Minnesota Veterinary Diagnostic lab.

The nucleic acid extraction kits QIAamp^®^ DSP Viral RNA Mini Kit (number 52906; SG-based) was purchased from Qiagen (Germantown, MD, USA).

### Filter Functionalization

The functionalization of Whatman filter paper was accomplished using TEOS (Tetraethyl Orthosilicate). A precise solution of TEOS was prepared by combining TEOS, ethanol, and water in a well-defined molar ratio of (0.5:25:8). To conduct the functionalization, the filter paper was introduced into a porcelain Buchner funnel equipped with a perforated plate. The solution was then suctioned through the filter, ensuring optimal polymer density and filter porosity. During this process, the hydrolysed TEOS underwent silanol condensation, establishing covalent bonds with the cellulose hydroxyl groups and resulting in a thin, uniform coating on the filter paper. This interaction between TEOS and cellulose generated a chemically bond and stable reactive layer.

### Propagation of viruses

Transmissible gastroenteritis virus (TGEV) was propagated and titrated in *Sus scrofa* testis (ST) cells. The cells were grown in Eagle’s Minimum Essential Medium supplemented with 8% fetal bovine serum (FBS), and 1×antibiotic-antimycotic. For virus propagation, the ST monolayer (80–90% confluency) was infected with the TGEV at 0.1 multiplicity of infection (m.o.i.) and maintained in Dulbecco’s Modified Eagle Medium supplemented with 2% FBS and 1× antibiotic-antimycotic. The infected cultures of TGEV were incubated in 5% CO_2_ incubators at 37°C for 3–5 days until cytopathic effects (CPE) were observed under an inverted microscope. The virus was harvested by only one cycle of freeze and thaw at −80°C, followed by centrifugation at 3,000 × g for 10 minutes to pellet and discard the cell debris for partial purification. The propagated virus stocks of TEGV were aliquoted and stored at −80°C until used in the experiment.

### Virus titration

The 50% tissue culture infective dose (TCID_50_) method was used to titrate the virus in its host cell monolayers. Serial 10-fold dilutions of the virus were prepared in the maintenance medium for the host cell described above. Confluent monolayers of the host cell prepared in 96-well plates were infected with 100μL of the virus dilution using 3 wells per dilution. The infected plates were incubated at 37° C in a 5% CO_2_ incubator. The cytopathic effects (CPE) of the infectious virus were observed under an inverted microscope after 5 days. The titer of the viruses was determined by a previously developed method and expressed as TCID_50_/mL.^22^

#### RT-qPCR for TGEV quantification.

PCR primers and probe set shown in **Table d** in the supplementary information were used.^23^ The RT-qPCR primers were designed to target a conserved 146 bp region (corresponding to the region between nucleotides 370 and 515 of the TGEV S gene open reading frame, with reference to the sequence of TGEV-GenBank accession no. KX900410.1). The reactions were performed using the AgPath-ID One-Step RT-PCR kit. The reaction mixture (25 μL) consisted of 5 μL of template RNA, 12.5 μL of 2× RT-PCR buffer, 1 μL 25× RT-PCR Enzyme Mix, 0.5 μL of 10 μM solutions of both TGEV-forward and reverse primers (200 nM final concentration), 0.30 μL of 10 μM probe solution (120 nM final concentration), and 5.20 μL of nuclease-free water. The RT-qPCR was performed in a QuantStudio-5 Real-Time PCR thermocycler system (Thermo Fisher Scientific-Applied BioSystems). The thermal cycling conditions of was 45°C/10 min for reverse transcription (RT), 95°C/15 min for Taq polymerase activation, and 45 PCR amplification cycles using a 94°C/1 s denaturation step and an annealing step of 58°C/45 s. In each run of RT-qPCR, standard curve samples and no template control were used as positive and negative controls, respectively.

#### Calibration curves of TGEV RT-qPCR.

The TGEV PCR standard/calibration curve was constructed for absolute quantification of viral genome copy number. Ten-fold serial dilutions of a 557 bp RT-PCR purified amplicon of TGEV S gene (including the 146 bp target sequence of the RT-qPCR primer/probe set) were used. The 557 bp TGEV S gene fragment was produced by RT-PCR reaction using an in-house developed primer set shown in **Table d** in the supplementary information. A 557 bp PCR amplicon with known copy number was used. Results were expressed as cycle threshold (Ct) values. The Ct values were used along with the standard curve to calculate the absolute genome copy number of TGEV, expressed as genome copies per mL.

### RT-PCR for SARS-CoV-2 quantification

RT-PCR using the standard US CDC primer-probe set for SARS-CoV-2 (N1 and N2 viral targets; human RNase P (RP) control) and operating under an FDA Emergency Use Authorization was done via the CLIA lab at the University of Minnesota Genomic Center.^17^ Samples were defined as positive for SARS-CoV-2 if either N1 or N2 exceeded the clinical thresholds (CT < 40 cycles); all samples required detection of the RP control (CT < 40) to meet criteria. Samples extracted using both the PC-based kit and the control SG-based kit were quantified through this method.

### Viral sample collection

Clinical nasopharyngeal swab specimens from routine COVID-19 testing were collected by a health care provider and transported in viral transport medium (VTM) or universal transport medium (UTM): fresh, refrigerated residual material from these collections were used for all extraction and molecular testing. All samples were obtained from the University of Minnesota Medical Center – Fairview system under Common Rule exemption.

### RNA extraction

Sample processing was conducted with both the SC- and PC-based kits. A graphical protocol diagram for the PC-based NA extraction kit is shown in **Figure b** in supplementary information. Briefly, a 100 μl aliquot of VTM/UTM was added to 560 μl of Buffer LB containing carrier RNA in a microcentrifuge tube and incubated at 56°C for 20 min to inactivate any virus present in the samples. After centrifugation (18,000×g; 1 min) the supernatant was centrifugally loaded onto a minispin column (850×g; 1 min), washed with buffer (WB; 500μL) and dried via extended centrifugation (18,000×g; 3 min). The bound RNA was eluted (EB, 1 min incubation; 4,500×g; 1 min) and the extracted samples were transferred for RT-PCR. Some of the samples were also extracted using the SG-based kit as control to compare the efficiency of the PC columns and buffers in extracting nucleic acids.

### Limit of detection (LOD)

To allow for quantitative determination of the limit of detection (LOD) of the assay using RNA extractions from each column type, a synthetic SARS-CoV-2 standard control manufactured by Exact Diagnostics (EDx; #COV019) at 200 cp/μL viral nucleic acid and 75 cp/μL human gDNA was diluted into the provided EDx negative control (human gDNA only; EDx: #COV000) and serial dilutions were prepared: 90 copies/μL (Cp/ μL), 45 Cp/ μL, 15 Cp/ μL, 5 Cp/ μL, 1.67 Cp/μL, and 0.56 Cp/μL. (These EDx controls are manufactured to serve as a synthetic spike-in source for assay validations: copy number is standardized via ddPCR by the manufacturer).^17^ LOD experiments were run in triplicate for most dilutions (Supplementary information **Table c**).

### Biosafety & Institutional Control

Extractions and processing of infectious viral samples were carried out under BSL-2 + conditions (standard BSL-2 conditions with the addition of some BSL-3 practices such as using extra personal protective equipment). All experimental protocols, including safety and regulatory protocols are approved by the University of Minnesota Institutional Biosafety Commission. All methods were carried out in accordance with relevant guidelines and regulations. Informed consent was obtained from all subjects and/or their legal guardian(s).

## Conclusions

The study reported here describes the development of a new kit for the extraction and purification of coronavirus RNA, including a new polymer composite column and assciated reganets. Comparison of the performance of the new kit with coventional silica gel columns and commercially available kits for SARS-CoV-2 RNA detection in clinical samples show that PC-based purification offers a significantly higher performance. PC-based kit was found to have similar sensitivity and 15.6% higher specificity compared to the SG-based kit, which is significant in preventing false positives in patient diagnosis. Furthermore, while both methods offer similar detection limit (1 copy/μL), the analytical precision of the RT-PCR assay is three times higher when using samples extracted and purified using the PC-based kit, thus improving both accuracy and result consistency across different experiments.

## Figures and Tables

**Table 1:** Comparison of the clinical specificity and sensitivity of nucleic acid extraction kits using silica gel (SG) or polymer composite (PC) –based purification.

	Preliminary study (16 samples)		Full scale study (66 samples)	
	SG-based purification	PC-based purification	SG-based purification	PC-based purification
**True Positives**	12	13	30	30
**False Positives**	0	0	5	0
**True negatives**	2	2	27	32
**False negatives**	2	1	2	2
**Sensitivity (%)**	85.71	92.86	93.75	93.75
**Specificity (%)**	100.00	100.00	84.38	100.00
**Accuracy (%)**	88.00	94.00	89.00	97.00

**Table 2:** Limits of detection, reproducibility and analytical precision of SG and PC kits.

	SG	PC
LOD (copies/ μL)	0.57	0.57
Analytical precision	0.09 Ct value per 1 RNA copy/μL	0.28 Ct value per 1 RNA copy/μL

## Data Availability

Raw data and datasets generated and/or analysed during the current study are available in supplementary information. Additional data or information is available under request by sending an email to the corresponding author at: aabbas@umn.edu.
